# Variation of soil nutrients and bacterial community diversity of different land utilization types in Yangtze River Basin, Chongqing Municipality

**DOI:** 10.7717/peerj.9386

**Published:** 2020-07-17

**Authors:** Yanlin Li, Chunmei Zeng, Meijun Long

**Affiliations:** Chongqing Key Laboratory of Environmental Materials & Remediation Technologies, College of Chemistry and Environmental Engineering, Chongqing University of Arts and Sciences, Chongqing, China

**Keywords:** Soil nutrients, Bacterial community diversity, Different land use types, Yangtze river basin

## Abstract

The diversity and community distribution of soil bacteria in different land use types in Yangtze River Basin, Chongqing Municipality were studied by using Illumina MiSeq analysis methods. Soil physical and chemical properties were determined, and correlation analyses were performed to identify the key factors affecting bacterial numbers and α-diversity in these soils. The results showed that the soil physical and chemical properties of different land use types decrease in the order: mixed forest (M2) > pure forest (P1) > grassland (G3) > bare land (B4). There were significant differences in bacterial diversity and communities of different land use types. The diversity of different land use types showed the same sequence with the soil physical and chemical properties. The abundance and diversity of bacterial in M2 and P1 soils was significantly higher than that in G3 and B4 soils. At phylum level, G3 and B4 soils were rich in only Proteobacteria and Actinobacteria, whereas M2 and P1 soils were rich in Proteobacteria, Actinobacteria and Firmicutes. At genus level, *Faecalibacterium* and *Agathobacter* were the most abundant populations in M2 soil and were not found in other soils. Pearson correlation analysis showed that soil moisture content, pH, AN, AP, AK and soil enzyme activity were significantly related to bacterial numbers, diversity and community distribution.

## Introduction

Soil is an important part of the ecosystem and plays an important role in maintaining the stability, material circulation and energy transformation of the terrestrial ecosystem (Wei et al., 2018a; Wei et al., 2018b). Soil microbes participate in soil organic matter decomposition, nutrient conversion and circulation, and have a wide range of functions in determining soil fertility, environmental monitoring and land use (Li et al., 2018a). Moreover, microbial biodiversity is highly essential in environmental pollution control and vegetation restoration in different land-use types (Wei et al., 2018a; Wei et al., 2018b).

Soil microbial community and diversity, which are important dynamic indicators of soil quality, can be affected by land-use conversions. Environmental conditions such as soil types, soil physical and chemical properties, and land-use patterns are major factors that influence soil microbial community diversity (Yang et al., 2018). The composition of soil bacterial communities, fungi, actinomycetes, nitrogen-fixing bacteria and ammonifying bacteria is significantly different in red soil, black soil, and gray desert soil (Xu et al., 2006). Saul-Tcherkas, Unc & Steinberger reported (2013) that low water content and soil nutrients reduce soil microbial diversity in the desert steppe ecosystem. Differences in the composition of the types of vegetation and the substances secreted by plant roots result in significant differences in soil microbial groups and numbers of different land-use types (Li et al., 2017a). Various factors influence the quantity and diversity of soil microbial communities. which also include land-use patterns (Zhen et al., 2019; Li et al., 2018c).

In recent years, studies on biodiversity have attempted to describe soil microbiomes in different ecosystems (Forest, desert, wetland, grassland and desert steppe ecosystem) to understand the dynamics of microbial interactions with the environment (Rao et al., 2016; Li et al., 2016; Zhang et al., 2017; Wei et al., 2018a; Li et al., 2018b). It was discovered that there are significant differences in soil microbial community structures between different ecosystems. A few studies have been reported on the soil microbial diversity in response to different land-use types. Zhao et al. (2013) compared five land-use types: natural forests, parks, agriculture, street greens and roadside trees, and found that soil properties and microbial diversities vary with land use. Garcha, Katyal & Sharma (2016) recorded a wide variation in the soil microbial population under different land-use systems in sub-mountainous zone of Punjab, and found that the total microbial population was maximum in the mixed forests followed by plantations and orchards and the least in the fodder crops and cultivated areas. Recent studies by Li et al. (2017b) demonstrated that the quantity and community distributions of isolated soil microorganisms were significantly different among different land-use types in typical semi-arid loess plateau biota in Northwest China. Soil microbial diversity and community structures under different land-use patterns need to be analyzed systematically with in-depth research.

The Yangtze River Basin in Chongqing is an important area of diverse land-use types in China. It has long been affected by natural and high-intensity human activities, and extreme soil erosion has made its ecosystem quite fragile. The ecological environment of this basin has gradually attracted attention (Yang, Liu & Dong, 2017). Much of the research on the Yangtze River Basin has been focused on soil and water conservation assessment (Fan et al., 2004; Liu et al., 2011; Kong et al., 2018). The study of soil microbial diversity in different land-use types is of great significance for clarifying the role of microbial communities in different environments and can provide theoretical guidance for rational land use. In this study, we described that the impact of land-use types on the abundance and diversity of soil microbes in the Yangtze River Basin, Chongqing Municipality by high-throughput sequencing technology. Our study provides a theoretical basis for the management of different land-use types in the region and provides a scientific basis for the comprehensive management of the basin and for monitoring and evaluation of the ecological environment.

## Materials and Methods

### Study site and soil sampling

The selected study sites belong to the Yangtze River Basin of Chongqing Municipality, with a mild climate and a subtropical monsoon humid climate. The annual mean temperature in the region is 16−18 °C and the normal year annual rainfall is 1,000–1,350 mm, more than 70% of which occurs from May to September. The types of landforms in the Yangtze River Basin are complex and diverse. The main soil types are purple soily, ellow loam, paddy soil, red soil, new soil, and mountain meadow soil. The area is part of the key environmental protection areas of the Yangtze River Basin because of the complex natural landforms, abundant precipitation, and erosive soils.

Based on, the characteristics of the Yangtze River Basin and the principle of vegetation division, the land types in the whole basin are divided into pure forest, mixed forest, grass, and bare land. The soil samples were collected randomly from different land-use types in the study area (N29°27^′^24.54^″^, E106°31^′^35.55^″^) in September 2018. Soil type in the research area is “red soil”. A total of 12 soil samples (three random samples per environment) were taken at four locations from pure forest (P1, Subtropical evergreen broad-leaved forest), mixed forest (M2, Subtropical mountain evergreen and deciduous broad-leaved mixed forest), grassland (G3), and bare land (B4). In each plot, the mixed soil samples (removing the surface litter layer) were collected from a depth of 0–20 cm by five-point sampling method and placed in individual sterile brown paper bags and stored at 4 °C for further studies. The fresh soil samples were divided into two parts, one part was passed through a 40 mesh sieve to determine the soil culturable bacterial number and diversity, while the other was naturally air dried to determine the soil nutrient.

### Soil properties

The physical and chemical characteristics of soils, including pH, moisture content, available nitrogen (AN), available phosphorus (AP) and available potassium (AK) were determined as described. Briefly, soil water content was estimated by drying the sample at 105 °C for 24 h (Lu, 2000). Soil pH was measured in 1:2.5 soil water suspensions (Lu, 2000). Available nitrogen was determined using the alkaline diffusion method (Page, Miller & Keeney, 1982). Soil available phosphorus was extracted with 0.5 mol/L NaHCO_3_ at a pH of 8.5, and P content was estimated colorimetrically by the molybdate method (Olsen, Cole & Watanable, 1954). Soil available potasssium was determined by flame spectrometry method (Lu, 2000). Soil enzyme activities were analyzed using standard methods (Chen et al., 2015). Catalase enzyme activity was determined by chemical titration. Soil urease activity was determined by estimating the amount of ammonia and carbonic acid produced during urea hydrolysis. Soil sucrase activity was determined using methods of 3,5-dinitrosalicylic acid colorimetry method.

### Determination of the viable count of culturable bacteria

The culturable bacterial counts were determined by plate counting method (Li et al., 2017a; Li et al., 2017b; Li et al., 2017c). Briefly, 10 g of soil sample was suspended in 90 mL of sterile distilled water and shaken for 30 min on a Thermostat oscillator. 0.1 mL of serial dilutions of the soil sample solutions were spread on Luria-Bertani (LB) agar plates and incubated at 30 °C for 24 h. Bacterial count was expressed as colony forming units per fresh weight (cfu/g) of soil. All experiments were carried out in triplicates.

### Metagenomic DNA isolation and community 16S rDNA gene amplification

DNA from all soil samples was extracted and purified using the E. Z. N. A.™ Mag-Bind Soil DNA Kit (D5625-01, Omega, USA) using 0.2 g of soil as per the manufacturer’s instructions and stored at −80 °C until further analysis. For bacterial 16S rRNA, PCR amplification was performed using the primers 16S 515F (5′-GTGCCAGCMGCCGCGGTAA-3′) and 16S 806R (5′-GGACTACHVGGGTWTCTAAT-3′) with genomic DNA as template. The PCR reaction conditions were as follows: initial denaturation at 94 °C for 2 min; denaturation at 94 °C for 30 s, annealing at 55 °C for 20 s, extension at 72 °C for 60 s, for 32 cycles; and a final extension at 72 °C for 10 min. PCR products were stored at −20 °C and analyzedon 1% gel using DL2000 marker for quality examination.

### Metagenomic sequencing

High-throughput sequencing was performed using the Illumina MiSeq platform at the Sangon Biotech Engineering Technology & Services Co., Ltd., Shanghai, China. The original sequence of MiSeq sequencing contains the barcode sequence primers and linker sequences. In order to ensure that the results of information analysis are more accurate and reliable, the raw reads were filtered using Cutadapt (V1.9.1, http://cutadapt.readthedocs.io/en/stable/) (Louca, Parfrey & Doebeli, 2016), and then compared with the species annotation database (http://github.com/torognes/vsearch/) (Martin, 2011) to delete the chimeric sequences and obtain clear reads (Rognes et al., 2016). Clean reads from all samples were clustered into OTUs (Operational Taxonomic Units) with 97% identity using Uparse software (Uparse v7.0.1001, http://www.drive5.com/uparse/) (Haas et al., 2011). The OTU sequences were annotated, using Mothur method and SSUrRNA database for species annotation analysis to obtain taxonomic information at each classification level (Edgar, 2013; Wang et al., 2007). After obtaining the sequencing results and calculation of operational taxonomic units (OTUs) matrix, statistical analysis was performed using alpha indices (Shannon, Simpson, Chao 1 and ACE), heatmap of genera, principal coordinate analysis (PCoA) and UPGMA. The alpha diversity index and UPGMA were calculated by using the Qiime software (Version 1.9.1) and Principal coordinate analysis (PCoA) was performed using the R package phyloseq.

### Statistical analyses

Statistical analysis of data was carried out using Origin 8.0 software (OriginLab Corp., USA). ANOVA with least-significant-difference (LSD) tests, cluster analysis, correlation analysis and principal component analysis were carried out using the SPSS 19.0 software (ver. 19.0; SPSS Inc., USA). For all analyses, the results were considered to be significant at *p* < 0.05.

## Results

### Soil physical and chemical properties

The basic physicochemical characteristics of soil samples from different land-use types are shown in Table 1. The soil pH ranged from 6.74–7.42, and with the M2 sample having the lowest pH. The moisture content ranged from 12.89% in sample B4 to 18.25% in sample M2 (*p* < 0.05). Significant differences in soil nutrients were observed in different land-use types. The soil available P (47.00 mg/kg), available K (112.61 mg/kg), available N (94.83 mg/kg) contents in M2 were significantly higher than those in P1, G3 and B4 soils. The soil catalase and sucrase activities were similar in the four samples, but the soil urease activity varied greatly, ranging from 0.7816 to 3.0474 mg/g (*p* < 0.05). The M2 soil sample had higher urease activity when compared with P1, G3 and B4.

### Number of soil cultivable bacteria in different land-use types

The number of culturable soil bacteria varied among different land-use types (Table 2). The culturable bacterial counts in P1, M2, G3 and B4 were 59. 3 ×10^5^, 85. 3 ×10^5^, 51. 3 ×10^5^ and 10. 0 ×10^5^ cfu/g respectively. The highest number of culturable bacteria was detected in M2 sample, while the lowest was found in B4.

### Abundance and diversity of members of the bacterial microbiota

**Figure 1:**
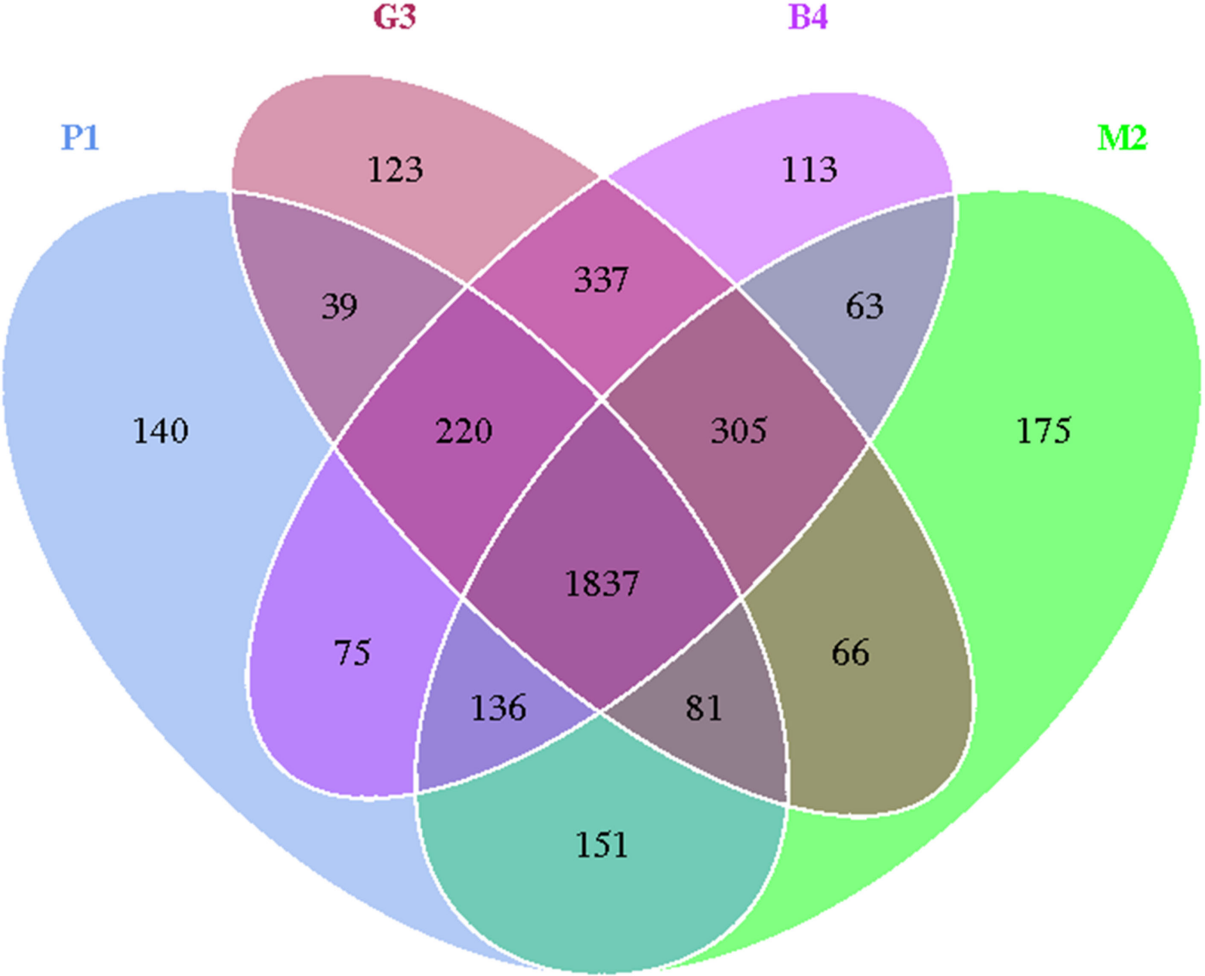
Venn diagram of OTU number in P1, M2, G3 and B4 soil samples. Venn diagrams showing OTUs specific to individual soil communities and those shared by multiple communities from pure forest (P1), mixed forest (M2), grassland (G3) and bare land (B4) soils.

Sequences with 97% or more similarity were classified as an operational classification unit (operational taxonomic units, OTU). As shown in Fig. 1, the sequences were grouped into 3861 OTUs, which reduced to 1837 after removing singletons, there was a similar trend in the P1, M2, G3 and B4 samples. Bacterial OTUs were 2679, 2814, 3008 and 3086 in P1, M2, G3 and B4 respectively.

The Good’ s coverage of P1, M2, G3 and B4 samples reached 99.1%, 98.7%, 99.1% and 99.0% respectively, that captured the majority of microbial diversity (Table 3). The Chao1/ACE index reflects the species richness information of the samples and the Shannon/Simpson index reflects the species diversity of microbes in a sample. The Chao1 and ACE scores ranged from 1867.768 to 2819.313 and from 1900.661 to 2542.059, respectively. The Shannon and Simpson scores ranged from 8.496 to 9.696 and 0.974 to 0.997, respectively (Table 3). The results showed that P1 had the largest Shannon index and the smallest Simpson index. One the other hand, M2 had maximum values for both the ACE index and the Chao1 index.

**Table 1:** The basic physicochemical characteristics of soil samples in study regions.

	Moisture content (%)	pH	Available P (mg/kg)	Available K (mg/kg)	Available N (mg/kg)	Catalase (mg/g)	Urease (mg/g)	Sucrase (mg/g)
P1	14.19 ± 0.75 a	7.35 ± 0.04 a	42.53 ± 1.34 a	41.98 ± 0.97 a	34.63 ± 0.81 a	0.5218 ± 0.002 a	1.6589 ± 0.1753 a	0.2282 ± 0.0000 a
M2	18.25 ± 1.11 a	6.74 ± 0.02 b	47.00 ± 2.65 b	112.61 ± 5.98 b	94.83 ± 7.00 a	0.5123 ± 0.011 a, b	3.0474 ± 0.6459 a	0.2173 ± 0.0013 b
G3	13.91 ± 0.00 a	7.42 ± 0.11 c	31.37 ± 2.43 c	39.14 ± 2.11 c	21.56 ± 2.80 b	0.5148 ± 0.004 a, b	1.0724 ± 0.0496 a	0.2236 ± 0.0014 c
B4	12.89 ± 0.79 b	7.08 ± 0.01 c	21.11 ± 2.87 c	27.63 ± 0.54 d	16.43 ± 0.81 c	0.5201 ± 0.003 b	0.7816 ± 0.1245 b	0.2276 ± 0.0014 c

**Notes.**

Results are expressed as the mean ± standard deviation of three independent tests. Different letters in the same column meant significant difference at 0.05 level.

### Comparison of soil bacterial communities in different land-use types

**Figure 2:**
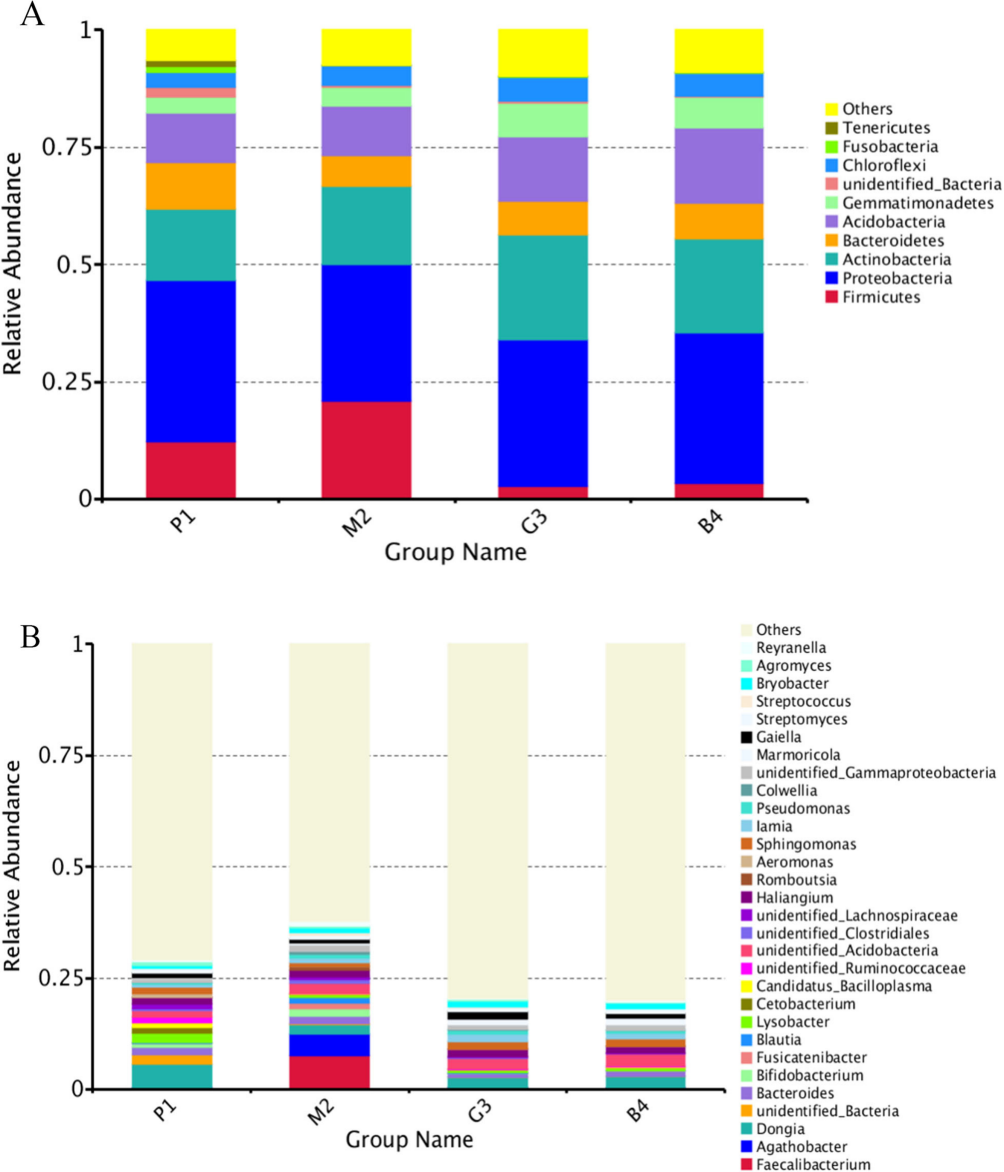
Relative abundance of bacterial at phylum and genus levels of P1, M2, G3 and B4. (A) Phylum level; (B) genus level (Analysis of amplified 16S rRNA gene sequences was performed in comparison with the RDP database at the 80% confidence level. The percentages of the phylogenetically classified sequences are plotted on the Y axis. P1: Pure forest; M2: Mixed forest; G3: Grassland; B4: Bare land).

**Figure 3:**
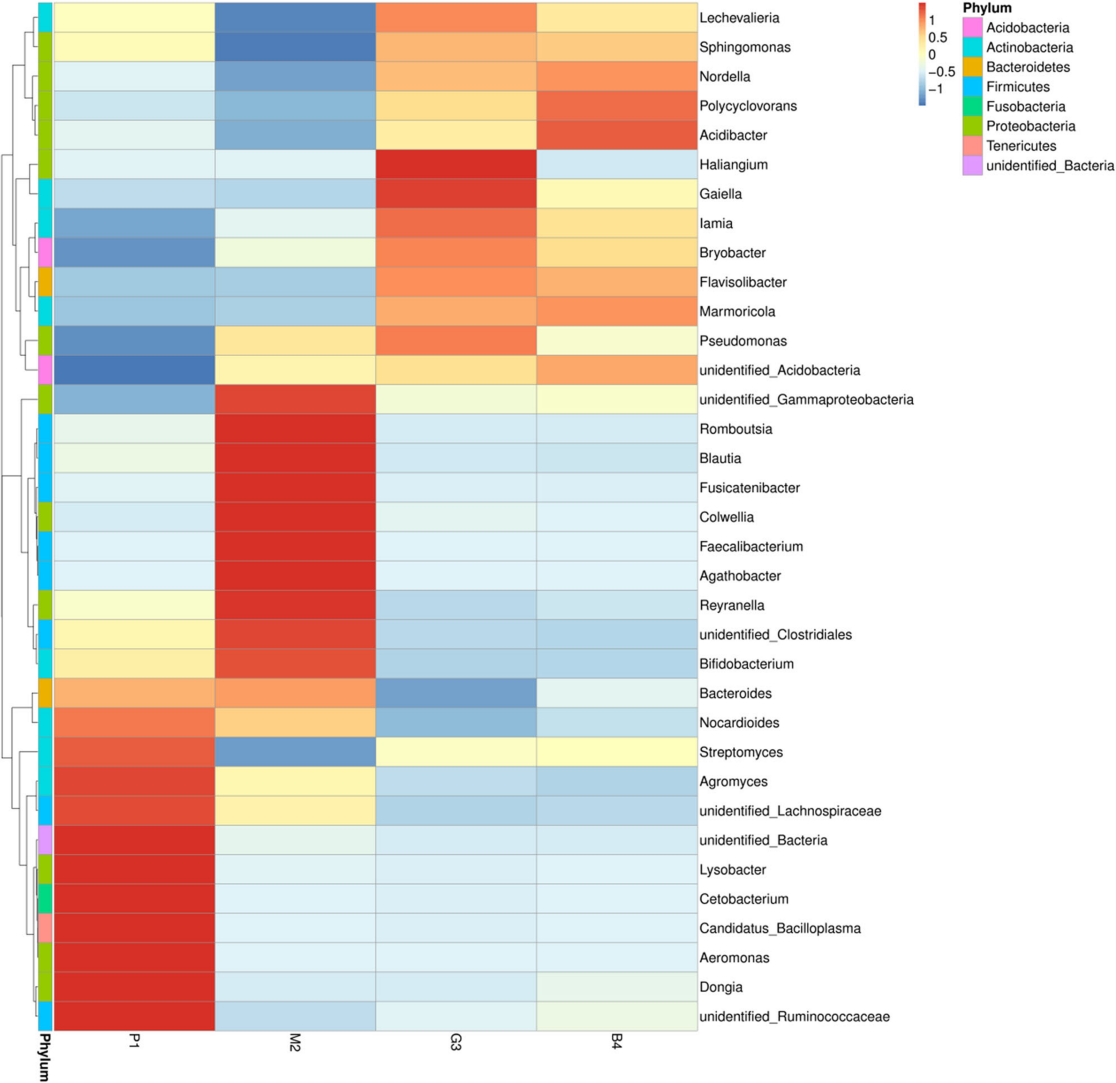
Based on the level of phylum cluster analysis. The sample information are plotted on the *X* axis, and the species annotation information are plotted on the *Y* axis. The cluster tree on the left is the species cluster tree. The corresponding value of the heat map is the *Z* value obtained by normalizing the relative abundance of the species in each row.

Nine bacterial phyla, Firmicutes, Proteobacteria, Actinobacteria, Bacteroidetes, Acidobacteria, Gemmatimonadetes, Chloroflexi, Fusobacteria and Tenericutes were identified in different land-use types analyzed (Fig. 2A). Among these Proteobacteria and Actinobacteria were the predominant phyla in P1, M2, G3 and B4 (Fig. 3). The vast majority of bacteria were classified as Proteobacteria and Actinobacteria in P1, and a similar pattern was observed in G3 and B4. The Firmicutes phylum was found to be abundantly distributed in P1 and M2, but was less abundant in G3 and B4 samples. Tenericutes were less abundant in P1 samples but not in M2, G3 and B4 samples. Heatmaps revealed that the bacterial community of P1 sample was more diverse than those of other samples (Fig. 3).

**Table 2:** Quantitative distribution of bacterial communities in different land use types.

Sampling point	Microbial populations (×10^5^ cfu/g)
P1	59.3 ± 8.5 a
M2	85.3 ± 3.1 b
G3	51.3 ± 6.1 b
B4	10.0 ± 2.0 c

**Notes.**

Results are expressed as the mean ± standard deviation of three independent tests. Different letters in the same column meant significant difference at 0.05 level.

**Table 3:** The statistics of data for Alpha diversity index.

Sample	Shannon index	Simpson index	Chao1 index	ACE index	Coverage
P1	9.696 ± 0.063 a	0.974 ± 0.040 a	1867.768 ± 724.337 a	1900.661 ± 739.888 a	0.991 ± 0.001 a
M2	9.590 ± 0.163 a	0.992 ± 0.003 a	2819.313 ± 920.797 a	2542.059 ± 35.515 a	0.987 ± 0.004 a
G3	8.603 ± 0.814 a	0.997 ± 0.000 a	2499.844 ± 32.549 a	2449.952 ± 371.808 a	0.991 ± 0.002 a
B4	8.496 ± 1.875 a	0.997 ± 0.000 a	2382.752 ± 88.036 a	2394.544 ± 69.038 a	0.990 ± 0.000 a

**Notes.**

Results are expressed as the mean ± standard deviation of three independent tests. Different letters in the same column meant significant difference at 0.05 level.

At the genus level, the diversity of the bacterial microbiota in P1, M2, G3 and B4 samples was higher, especially in the M2 samples (Figs. 2B, 3). Twenty-four bacterial genera were detected with higher relative abundance in P1, M2, G3 and B4. *Dongia*, *Aeromonas*, *Candidatus_Bacilloplasma*, *Cetobacterium*, *Lysobacter* and *Agromyces* were the predominant genera in P1 samples. In M2 samples, majority of the bacterial community belonged to eight genera, *Romboutsia*, *Blautia*, *Fusicatenibacter*, *Colwellia*, *Faecalibacterium*, *Agathobacter*, *Reyranella* and *Bifidobacterium*. *Haliangium* and *Gaiella* were the predominant genera in G3 samples, while *Acidibacter* and *Polycyclovorans* were the predominant genera in B4 samples. Other major genera were *Bryobacter*, *Streptococcus*, *Streptomyces*, *Marmoricola*, *Pseudomonas*, *lamia*, *Sphingomonas* and *Bacteroides*. *Dongia* was less abundant in M2, G3 and B4, and was found to be higher in P1 samples. *Faecalibacterium* and *Agathobacter* were highly abundant in M2 samples but were rarely detected in P1, G3 and B4 samples. The bacterial microbial community thus varied greatly, and the abundance of each genus varied among soil samples from different land-use types.

**Figure 4:**
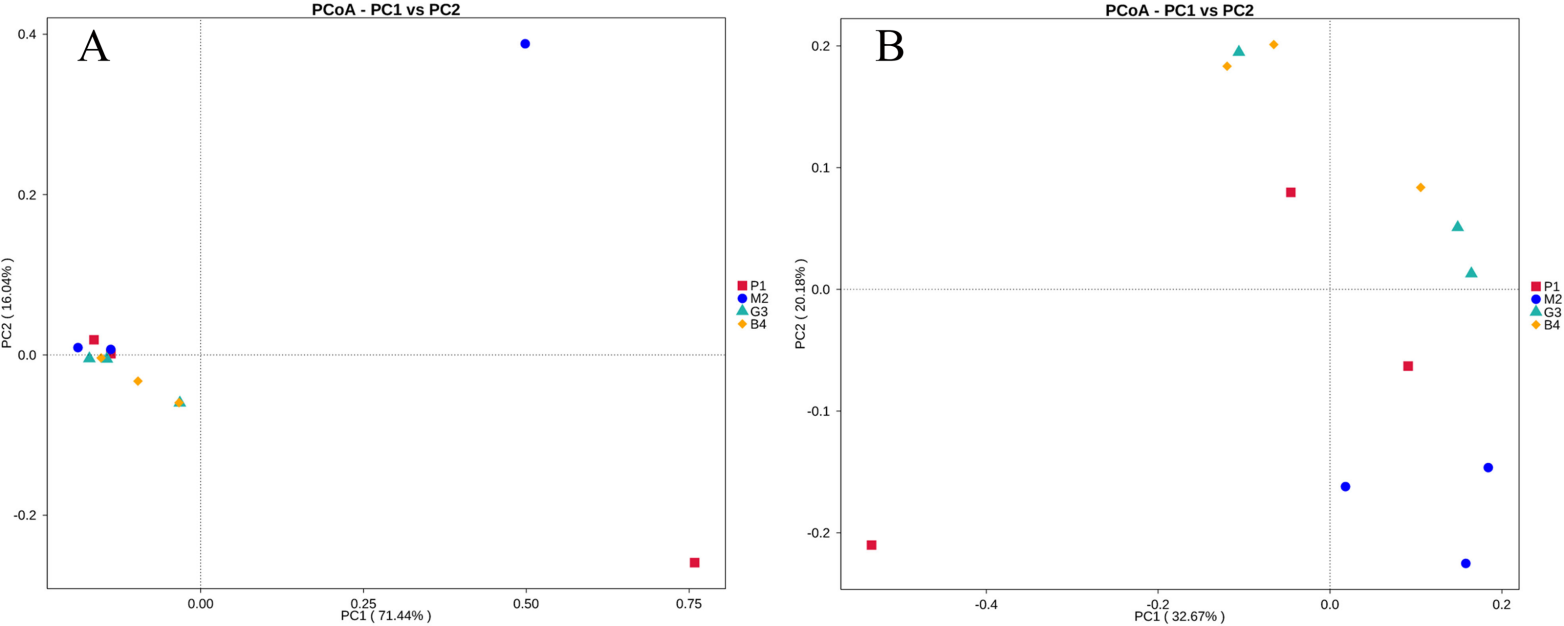
PCoA analyses of bacterial microbial communities of P1, M2, G3 and B4 samples. (A) PCoA analysis based on Weighted Unifrac distance; (B) PCoA analysis based on Unweighted Unifrac distance. (The abscissa represents one principal component, the ordinate represents another principal component, and the percentage represents the contribution value of the principal component to the sample difference. Each point in the figure represents a sample, and the samples of the same group are represented by the same color.)

**Figure 5:**
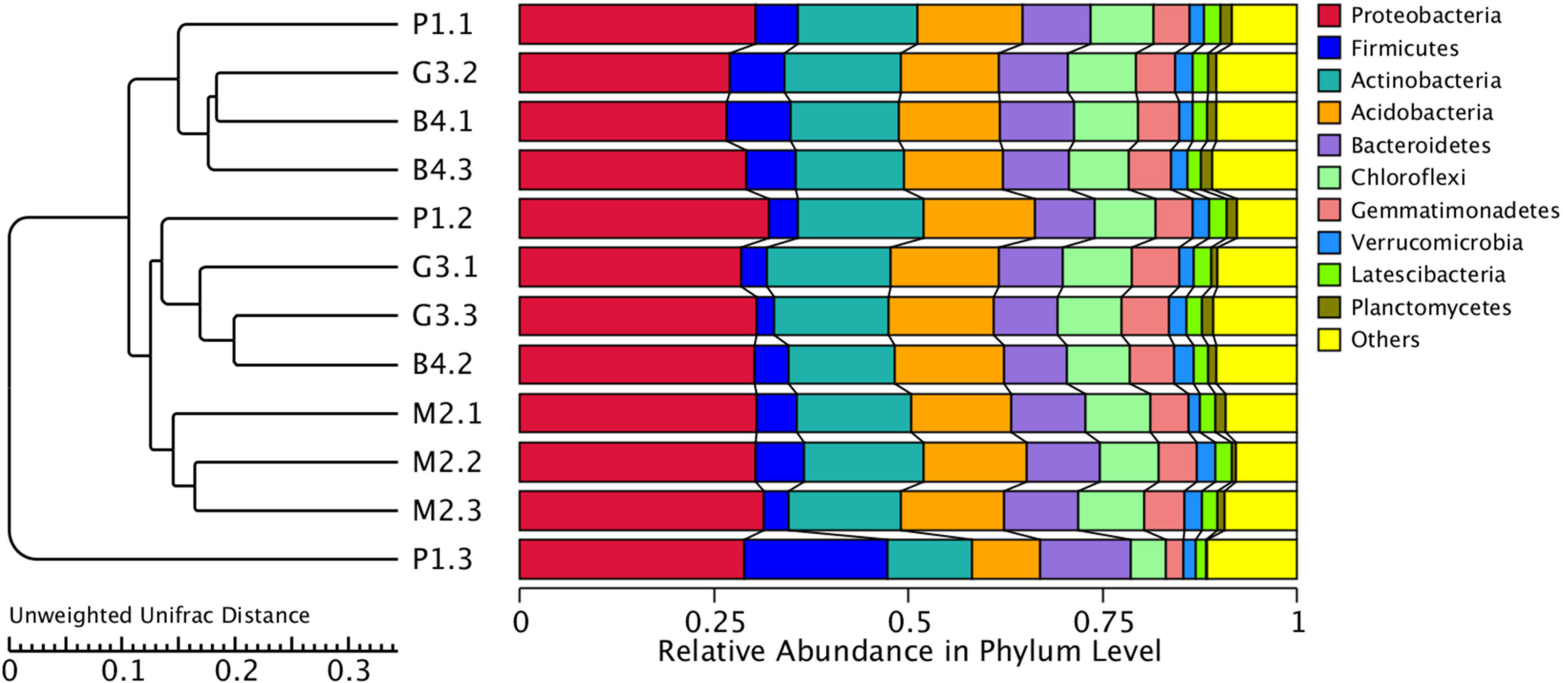
Cluster analysis of bacterial microbial communities of P1, M2, G3 and B4 samples. The UPGMA cluster tree structure is on the left, and the relative abundance distribution of each sample at the gate level is on the right.

PCoA and UPGMA analyses were performed to evaluate similarities in the bacterial communities of P1, M2, G3 and B4. One weighted (PC1 = 71.44%, PC2 = 16.04%) and another weighted (PC1 = 32.67%, PC2 = 20.18%) PCoA of bacterial communities was performed to analyze the community structure similarity (Fig. 4). The UPGMA clustering tree was built by using unweighted group averaging method (Fig. 5). The branch length of the samples reflected their similarity. The results showed that there was higher similarity among the community structures of all soils, although some differences existed in the study region of different land-use types.

### Correlations between microbial diversity and environmental factors

To further estimate the effects of environmental factors on microbial quantity and diversity, soil environmental factors such as moisture content, pH, available P, available N, available K, catalase, urease and sucrase in the samples were analyzed by correlation analysis using SPSS 19.0. These analyses demonstrated that environmental factors were strongly correlated with the microbial quantity and diversity (*p* < 0.01). The canonical correlation of the eight soil properties with the culturable bacterial numbers and α-diversity of the P1 soil are listed in Table 4. The bacterial numbers were significantly positively correlated with catalase and sucrase (*P* < 0.05), with correlation coefficients of 0.823 and 0.993 respectively. The Shannon index was negatively correlated (−0.806) and Simpson index was positively correlated (0.992) with pH. The Chao1 index and ACE index were significantly positively correlated with catalase, with correlation coefficients of 1.000 and 0.999 respectively (*P* < 0.01). The data in Table 5 indicate that moisture content (−0,926), catalase (0.957) and urease (0.851) had significant effects on microbial quantity. And available K (−1.000) and available N (−0.999) were negatively correlated with bacterial diversity and abundance in the M2 sample. Available K and moisture content are dominant factors that affect the number of culturable bacteria, and the α-diversity index was primarily positively affected by available K and urease in the G3 sample (Table 6). The culturable bacterial quantity and α-diversity index showed a significantly negative correlation with sucrase (−0.971) and available P (−0.953) in the B4 sample (Table 7).

**Table 4:** Pearson correlation coefficients for the relationships between the soil properties and bacterial diversity of P1.

	Number	Shannon	Simpson	Chao1	ACE
pH	−0.229	−0.806	0.992	0.359	0.397
Moisture	0.233	−0.453	0.829	0.740	0.767
AN	−0.645	0.005	−0.489	−0.963	−0.974
AP	0.282	−0.408	0.799	0.773	0.799
AK	0.207	−0.477	0.843	0.722	0.750
Catalase	0.823	0.257	−0.244	1.000^**^	0.999^*^
Urease	−0.672	−0.031	0.458	−0.972	−0.981
Sucrase	0.988	0.651	−0.201	0.904	0.885

**Notes.**

* Significant correlation at 0.05 level (both sides).

** Significantly correlated at 0.01 level (both sides).

**Table 5:** Pearson correlation coefficients for the relationships between the soil properties and bacterial diversity of M2.

	Number	Shannon	Simpson	Chao1	ACE
pH	0.500	0.996	−0.849	−0.312	−0.967
Moisture	−0.926	−0.045	0.409	0.984	0.123
AN	0.217	0.977	−0.968	−0.012	−0.999^*^
AP	0.590	−0.486	0.827	−0.743	−0.626
AK	−0.421	−1.000^**^	0.892	0.226	0.986
Catalase	0.957	0.139	−0.321	−0.996	−0.029
Urease	0.851	0.833	−0.496	−0.726	−0.729
Sucrase	−0.189	0.812	−0.988	−0.386	−0.899

**Notes.**

* Significant correlation at 0.05 level (both sides).

** Significantly correlated at 0.01 level (both sides).

**Table 6:** Pearson correlation coefficients for the relationships between the soil properties and bacterial diversity of G3.

	Number	Shannon	Simpson	Chao1	ACE
pH	−0.137	0.584	−0.052	0.444	0.881
Moisture	0.230	0.838	−0.410	0.090	0.651
AN	−0.327	−0.889	0.500	−0.011	−0.571
AP	0.039	0.717	−0.227	0.280	0.785
AK	0.946	0.909	−0.990	−0.794	−0.294
Catalase	−0.886	−0.322	0.782	0.987	0.897
Urease	−0.540	0.190	−0.371	0.779	0.998^*^
Sucrase	−0.047	0.655	−0.143	0.361	0.835

**Notes.**

* Significant correlation at the 0.05 level (both sides).

**Table 7:** Pearson correlation coefficients for the relationships between the soil properties and bacterial diversity of B4.

	Number	Shannon	Simpson	Chao1	ACE
pH	0.866	0.489	−0.500	0.862	0.634
Moisture	−0.513	0.865	−0.859	−0.520	−0.782
AN	−0.866	0.511	−0.500	−0.870	−0.987
AP	−0.290	−0.953	0.957	−0.282	0.066
AK	0.460	−0.894	0.888	0.467	0.743
Catalase	−0.812	−0.573	0.583	−0.807	−0.555
Urease	0.628	0.770	−0.778	0.622	0.314
Sucrase	−0.971	−0.228	0.240	−0.969	−0.824

## Discussion

Previous studies have demonstrated that bacterial abundance and diversity can be influenced by factors such as soil type, organic matter content, soil properties and plant species (Hu et al., 2019; Cai et al., 2018; Mohamed & Abdelmajid, 2017; Li et al., 2017a; Li et al., 2017b; Li et al., 2017c). It is commonly believed that different land-use types impact bacterial quantity in soils (Song et al., 2013). Song et al. (2013) found that primary forest and farmland had the highest quantity of soil microbial populations, while forest plantation had the lowest. In this study, we observed that soil characteristics and soil enzyme activity varied in different land-use types and actively influences bacterial quantity and diversity in the Yangtze River Basin, Chongqing Municipality. Our results showed that the bacterial numbers in B4 samples (10.0 × 10^5^ cfu/g) were significantly lower than those in P1, M2, G3 samples. We found that coverage by plants was a key factor influencing the number of soil bacteria, plant residues are considered to be important organic components of soil microbial growth (Wang et al., 2018).

Land usage has a significant impact on soil properties including moisture content, pH, soil nutrients and soil enzyme activity (Kiflu, 2013). Qi et al. (2018) investigated the changes in soil physical, chemical properties and microbial biomass dynamics due to land use changes in fixed desertified land and showed that changing shrubland to arable land and nursery garden significantly increased BD, SOM, CEC, TN and available N, P and K. Muche, Kokeb & Molla (2015) found variations in soil physicochemical properties observed in the grazing field, cultivated land, and plantation forest land-use types in Alket Wonzi Watershed, Farta district, Northwest Ethiopia. There were significant differences in soil carbon, nitrogen and phosphorus contents due to land use changes in Brazil (Groppo et al., 2015). In our study, the soil moisture content, soil nutrients and soil urease of different land-use types decreased in the order: M2>P1>G3>B4. For the M2 and P1 samples, soil nutrient accumulation was mainly from decomposition of litters. However, in the B4 sample, the accumulation of litter is very lower, which induces slower accumulation of soil nutrients (Qi et al., 2015; Qi et al., 2018). Liu et al. (2010) and Alabi et al. (2019) showed that land use type and species of vegetation planted differently influence the physical and chemical properties of the soil. Mojiri, Hamidi & Amin (2012) also noted that land-use change has a significant effect on many of these soil quality indicators.

Chang in land use not only affects soil physical and chemical properties but also affects soil microbe populations. Hu et al. (2019) reported that soil microbes are more susceptible to soil organic carbon, organic nitrogen and nutrient (phosphate, ammonium) content. Yang, Liu & Dong (2017) reported that the abundance and diversity of different land-use types in the Jialing River, Sichuan Province followed the sequence: mixed forest >broad-leaved forest >coniferous forest >shrub >meadow >bare land, with similar trends for change of soil physical and chemical properties. Our observation of significant differences in bacterial community structures in different land-use type soils support this finding. The statistics for alpha diversity index showed that bacterial diversity followed the order P1>M2>G3>B4, while the bacterial abundance followed the order M2>P1>G3>B4. This is highly consistent with the trends of soil physical and chemical properties in different land-use patterns. Moreover, correlation analysis also demonstrated that the number, diversity and abundance of soil bacteria varied, according to changing soil physical and chemical properties caused by changes in land-use types. In this study, higher moisture content, AN, AP and AK resulted in the highest level of bacterial diversity in B4 samples.

Different land-use types had also had an important impact on soil bacterial community composition, which may change the soil function and ecological processes (Yuan et al., 2015; Wang et al., 2018). Previous studies have reported that soils showed different bacterial diversity in different land-use types. Rampelotto et al. (2013) analyzed the soil bacterial communities under different land-use types in Cerrado by high throughput pyrosequencing of 16S rRNA genes. The results showed that more bacterial groups as Acidobacteria, Actinobacteria and Chloroflexi were present in sugarcane fields compared to natural forests. Song et al. (2013) compared the composition of bacterial communities in farmland, grassland, scrub, forest plantation, secondary forest and primary forest land-use types in karst hills. The forest plantation, secondary forest and primary forest land-use types had a higher proportion of soil bacterial diversity compared to others. Lynn et al. (2017) noted that Proteobacteria and Actinobacteria were most abundant in wetland, grassland and tea plantation soils, whereas Chloroflexi was dominant in forest soils. Our study also demonstrated that the composition of soil bacterial communities varied under different land-use soils (Figs. 2, **3). In this study, Proteobacteria, Actinobacteria and Firmicutes were found to be the most abundant bacterial genera in P1 and M2 soil samples. G3 and B4 soils were rich only in Proteobacteria and Actinobacteria. The results suggested that Proteobacteria and Actinobacteria adapt better to all environments, and Firmicutes prefer nutrient rich environments (pure forest and mixed forest). However, the bacterial diversity was highest in M2 soil compared with other soils; *Faecalibacterium* and *Agathobacter* were especially dominant, and were rarely detected in P1, G3 and B4 samples. This finding is in accordance with that of Goldfarb et al. (2011), who found that the bacterial diversity increased with an increase in soil nutrients (AN, AP, AK). Correlation analysis in this study also showed that there was a strong correlation between soil bacterial alpha diversity and soil physical and chemical properties (Tables 4–7), and also that there were differences in bacterial communities under different land use patterns.

## Conclusions

Our data highlight that land use has strong effects on soil properties, soil bacterial abundance, diversity and community composition. Correlation analysis showed that pH, water content, AN, AP, AK and soil enzymes were significantly related to bacterial count and diversity. The bacterial numbers were higher in M2 soil than in other land-use soils. The highest bacterial diversity was observed in M2 soil and that in B4 soil was lowest because of poor nutrient availability. Furthermore, the results revealed that *Faecalibacterium* and *Agathobacter* were dominant in M2 soil and different when compared with P1, G3 and B4 soils. Our study not only lays the foundation for understanding the relationships between microbes, plants and soil, but also provides a theoretical basis for land management.

##  Supplemental Information

10.7717/peerj.9386/supp-1 Supplemental Information 1 Raw data for Tables 1–3

10.7717/peerj.9386/supp-2 Supplemental Information 2 Sequences of 16 S rDNA

## Additional Information and Declarations

### Data Availability

The following information was supplied regarding data availability: The raw measurements are available in the Supplemental File. The raw sequence reads are available at BioProject: PRJNA610448; BioSample: SAMN14297879, SRX8527246.
