# Supplementary material for: Variation of soil nutrients and bacterial community diversity of different land utilization types in Yangtze River Basin, Chongqing Municipality
Source: PeerJ. 2020 Jul 17;8:e9386. doi: 10.7717/peerj.9386 (PMC7370933; doi:10.7717/peerj.9386)
Supplement: Supplemental Information 1 [file peerj-08-9386-s001.docx]

| Moisture content (%) | 1 | 2 | 3 |
| --- | --- | --- | --- |
| P1 | 14.21 | 14.94 | 13.44 |
| M2 | 19.53 | 17.67 | 17.55 |
| G3 | 14.00 | 13.90 | 13.83 |
| B4 | 12.88 | 12.11 | 13.69 |
| pH | 1 | 2 | 3 |
| P1 | 7.32 | 7.39 | 7.31 |
| M2 | 6.74 | 6.76 | 6.73 |
| G3 | 7.54 | 7.38 | 7.37 |
| B4 | 7.09 | 7.08 | 7.08 |
| Available P (mg/kg) | 1 | 2 | 3 |
| P1 | 44.04 | 42.08 | 41.47 |
| M2 | 44.49 | 46.75 | 49.77 |
| G3 | 31.67 | 33.63 | 28.80 |
| B4 | 18.70 | 24.28 | 20.36 |
| Available K (mg/kg) | 1 | 2 | 3 |
| P1 | 41.48 | 41.36 | 43.10 |
| M2 | 110.84 | 107.72 | 119.28 |
| G3 | 38.58 | 37.37 | 41.48 |
| B4 | 27.38 | 27.26 | 28.25 |
| Available N (mg/kg) | 1 | 2 | 3 |
| P1 | 34.16 | 34.16 | 35.56 |
| M2 | 95.76 | 101.36 | 87.36 |
| G3 | 18.76 | 21.56 | 24.36 |
| B4 | 15.96 | 15.96 | 17.36 |
| Catalase (mg/g) | 1 | 2 | 3 |
| P1 | 0.5238 | 0.5225 | 0.5190 |
| M2 | 0.5130 | 0.5010 | 0.5230 |
| G3 | 0.5175 | 0.5106 | 0.5164 |
| B4 | 0.5172 | 0.5218 | 0.5213 |
| Urease (mg/g) | 1 | 2 | 3 |
| P1 | 1.5546 | 1.5669 | 1.8613 |
| M2 | 2.5730 | 3.7877 | 2.7816 |
| G3 | 1.1252 | 1.0270 | 1.0638 |
| B4 | 0.8920 | 0.8061 | 0.6466 |
| Sucrase (mg/g) | 1 | 2 | 3 |
| P1 | 0.2287 | 0.2281 | 0.2278 |
| M2 | 0.2181 | 0.2181 | 0.2158 |
| G3 | 0.2252 | 0.2230 | 0.2226 |
| B4 | 0.2260 | 0.2280 | 0.2288 |
| Microbial populations （×10^4^ cfu/g） | 1 | 2 | 3 |
| P1 | 690 | 560 | 530 |
| M2 | 820 | 880 | 860 |
| G3 | 500 | 580 | 460 |
| B4 | 120 | 100 | 80 |
| Shannon index | 1 | 2 | 3 |
| P1 | 9.758 | 9.633 | 9.696 |
| M2 | 9.571 | 9.761 | 9.437 |
| G3 | 9.111 | 9.034 | 7.665 |
| B4 | 9.554 | 6.331 | 9.602 |
| Simpson index | 1 | 2 | 3 |
| P1 | 0.997 | 0.928 | 0.996 |
| M2 | 0.993 | 0.994 | 0.988 |
| G3 | 0.997 | 0.998 | 0.996 |
| B4 | 0.997 | 0.997 | 0.997 |
| Chao1 index | 1 | 2 | 3 |
| P1 | 2465.023 | 2076.219 | 1062.061 |
| M2 | 3873.958 | 2175.113 | 2408.869 |
| G3 | 2518.276 | 2462.262 | 2518.993 |
| B4 | 2470.364 | 2383.594 | 2294.298 |
| ACE index | 1 | 2 | 3 |
| P1 | 2485.543 | 2147.542 | 1068.897 |
| M2 | 2539.216 | 2508.051 | 2578.910 |
| G3 | 2838.494 | 2097.511 | 2413.852 |
| B4 | 2445.122 | 2422.619 | 2315.892 |
| Coverage | 1 | 2 | 3 |
| P1 | 0.986 | 0.991 | 0.995 |
| M2 | 0.983 | 0.986 | 0.991 |
| G3 | 0.989 | 0.992 | 0.991 |
| B4 | 0.991 | 0.990 | 0.990 |
